# Bartter Syndrome: A Systematic Review of Case Reports and Case Series

**DOI:** 10.3390/medicina59091638

**Published:** 2023-09-11

**Authors:** Rakhtan K. Qasba, Anna Carolina Flumignan Bucharles, Maria Victoria Ferreira Piccoli, Pranjal Sharma, Akshat Banga, Balakrishnan Kamaraj, Faisal A. Nawaz, Harshadayani Jagadish Kumar, Mahika Afrin Happy, Ruman K. Qasba, Gowthami Sai Kogilathota Jagirdhar, Mohammad Yasir Essar, Piyush Garg, Shiva Teja Reddy, Kaanthi Rama, Salim Surani, Rahul Kashyap

**Affiliations:** 1 Green Life Medical College and Hospital, Dhaka 1205, Bangladesh; 2 Department of Medicine, Faculty of Health Sciences, Universidade Positivo, R. Professor Pedro Viriato Parigot de Souza, Curitiba 5300, Brazil; 3 MercyOne Hospital, Clinton, IA 52732, USA; 4 Sawai Man Singh Medical College, Jaipur 302004, Rajasthan, India; 5 Madurai Medical College, Madurai 625020, Tamil Nadu, India; 6 Emirates Health Services, Al Amal Psychiatric Hospital, Dubai 345055, United Arab Emirates; 7 Sapthagiri Institute of Medical Sciences, Banglore 560090, Karnataka, India; 8 Sher-I-Kashmir Institute of Medical Sciences, Srinagar 190001, Jammu and Kashmir, India; 9 Department of Medicine, Saint Michaels Medical Center, Newark, NJ 07102, USA; 10 Department of Global Health, McMaster University, Hamilton, ON L8S 4L8, Canada; 11 JJM Medical College, Davanagere 577004, Karnataka, India; 12 Gandhi Medical College, Secunderabad 500025, Telangana, India; 13 Department of Medicine & Pharmacology, Texas A&M University, College Station, TX 79016, USA; 14 Critical Care Medicine, Department of Anesthesiology, Mayo Clinic, Rochester, MN 55905, USA

**Keywords:** Bartter syndrome, salt-losing tubulopathies, autosomal-recessive tubulopathies

## Abstract

*Background and Objectives*: Bartter syndrome (BS) is a rare group of autosomal-recessive disorders that usually presents with hypokalemic metabolic alkalosis, occasionally with hyponatremia and hypochloremia. The clinical presentation of BS is heterogeneous, with a wide variety of genetic variants. The aim of this systematic review was to examine the available literature and provide an overview of the case reports and case series on BS. *Materials and Methods*: Case reports/series published from April 2012 to April 2022 were searched through Pubmed, JSTOR, Cochrane, ScienceDirect, and DOAJ. Subsequently, the information was extracted in order to characterize the clinical presentation, laboratory results, treatment options, and follow-up of the patients with BS. *Results*: Overall, 118 patients, 48 case reports, and 9 case series (*n* = 70) were identified. Out of these, the majority of patients were male (*n* = 68). A total of 21 patients were born from consanguineous marriages. Most cases were reported from Asia (73.72%) and Europe (15.25%). In total, 100 BS patients displayed the genetic variants, with most of these being reported as Type III (*n* = 59), followed by Type II (*n* = 19), Type I (*n* = 14), Type IV (*n* = 7), and only 1 as Type V. The most common symptoms included polyuria, polydipsia, vomiting, and dehydration. Some of the commonly used treatments were indomethacin, potassium chloride supplements, and spironolactone. The length of the follow-up time varied from 1 month to 14 years. *Conclusions*: Our systematic review was able to summarize the clinical characteristics, presentation, and treatment plans of BS patients. The findings from this review can be effectively applied in the diagnosis and patient management of individuals with BS, rendering it a valuable resource for nephrologists in their routine clinical practice.

## 1. Introduction

Bartter syndrome (BS) is a rare group of autosomal-recessive salt-losing tubulopathies characterized by impaired transport mechanisms in the thick ascending limb of the loop of Henle (TAL), resulting in pronounced salt wasting. It was first reported in 1988 by Frederic C. Bartter as a novel syndrome [1], marked by hypokalemic metabolic alkalosis with hyperreninemic hyperaldosteronism in a normotensive patient [2].

BS is classified into five types, based on distinct genotypic and phenotypic manifestations. Although all of the types involve defective salt reabsorption along the TAL, the phenotypes often overlap, with molecular patterns associated with specific genes [2].

In Type I BS, the symptoms typically appear at birth, characterized by severe salt wasting, hyposthenuria, elevated PGE2 production, and failure to thrive. Some symptoms may arise in utero, leading to polyhydramnios and premature birth. It is considered to be the most common form, often caused by mutations in the SLC12A1 gene, affecting the NKCC2 cotransporter in TAL [2,3,4]. Type II BS is a subtype that is also known as antenatal Bartter syndrome, which is primarily linked to mutations in the KCNJ1 gene, affecting the ROMK channel. It presents prenatally or shortly after birth with polyhydramnios, premature delivery, and severe dehydration [4,5]. Type III BS results from CLCNKB gene mutations, impacting the chloride channel ClC-Kb in the kidneys’ distal tubules. It exhibits milder symptoms than the classic form, often appearing in childhood or adolescence [2]. Type IV is sub-grouped into two types: Type IVa and Type IVb. BSND gene mutation causes Type IVa BS, leading to defective barttin insertion in the CLC-Kb and CLC-Ka channels within the kidneys’ loop of Henle and the inner ear, disrupting salt transport. Conversely, Type IVb involves mutations in both the CLCNKA and the CLCNKB genes, resulting in impaired functioning of two chloride channels, severe salt wasting, and deafness. Both BSND and CLCNKA/CLCNKB mutations are associated with polyhydramnios, preterm delivery, and impaired urinary concentration [2]. Type V is a newly discovered one, with a usual X-linked recessive inheritance pattern, contrary to the other types, which are autosomal-recessive. Here, a CASR gene mutation leads to hypercalciuria, in addition to the main underlying symptoms seen in BS patients.

Salt supplementation, NSAIDs, and aldosterone antagonists are considered viable options for treating BS [2,6]. Prenatally, amniocentesis and/or indomethacin therapy have been reported to be effective. Due to the relatively new discovery and rarity of this disease, the treatment options are very much limited, with no curative options available, thus rendering the management of these patients entirely symptomatic. Adding to this, the literature covering the clinical, epidemiological, and therapeutic interventions for this syndrome is very limited. Therefore, we aim to conduct a systematic review of the available case reports and case series reporting BS.

## 2. Materials and Methods

This review has been reported in accordance with the PRISMA (Preferred Reporting Items for Systematic Reviews and Meta-Analyses) statement, as indicated in the PRISMA checklist [7], and registered with PROSPERO (IDCRD42022351227; www.crd.york.ac.uk/prospero accessed on 15 June 2023).

### 2.1. Search and Selection

An electronic search of five bibliographic databases, including Pubmed, Cochrane, DOAJ, Science Direct, and JSTOR, was conducted for case reports/series regarding BS published in English between April 2012 and 2022, i.e., in the last 10 years. Using a combination of keywords and medical subject headings (MeSH), we used vocabulary related to “Bartter” OR “Bartter syndrome” OR Bartter* OR “Sodium-Potassium-Chloride Symporters” AND “Tubulopathy”.

Two authors (R.Q. and A.F.) were involved in the study selection. After removing duplicates using Zotero [8], title and abstract screening was performed independently by eight authors (R.Q., A.F., V.P., A.B., B.K., H.J., S.T., and K.R.) using Microsoft Excel. Studies meeting the inclusion criteria were retrieved and screened for the full text. Conflicts between two authors screening the same studies were resolved among the authors. If a consensus was not reached, an additional third arbiter was added to solve the disagreements.

### 2.2. Data Extraction

Four independent authors (A.F., V.P., M.H., and A.B.) performed data extraction from all of the included studies into a pre-piloted data extraction form in Microsoft Excel. A fifth author (R.Q.) independently extracted data for validation. The following was extracted from each study:

*General information*: Author, title, DOI, year of publication, and journal. 

*Study characteristics*: Country, number of cases, journal, study conclusion, and study design.

*Participant characteristics*: Gender, age, birth weight, BS type, associated genetic mutation, patient presentation, family history, consanguinity, head circumference, Apgar score, laboratory studies, complications, treatment, follow-up, and conclusion.

### 2.3. Synthesis of Results

Descriptive statistics were used to calculate simple frequency, percentage, and proportion from the extracted data, reporting continuous data points as median (IQR) or mean (+/− SD), categorical variables as percentages, and outcomes as a number and percentage.

### 2.4. Assessment of Risk of Bias

Two authors (R.Q. and A.F.) independently performed the quality assessment of the included studies using the Joanna Briggs Institute (JBI) critical appraisal checklist for case reports and series [9]. Any discrepancies were resolved through discussion.

## 3. Results

### 3.1. Study Selection

**Figure 1:**
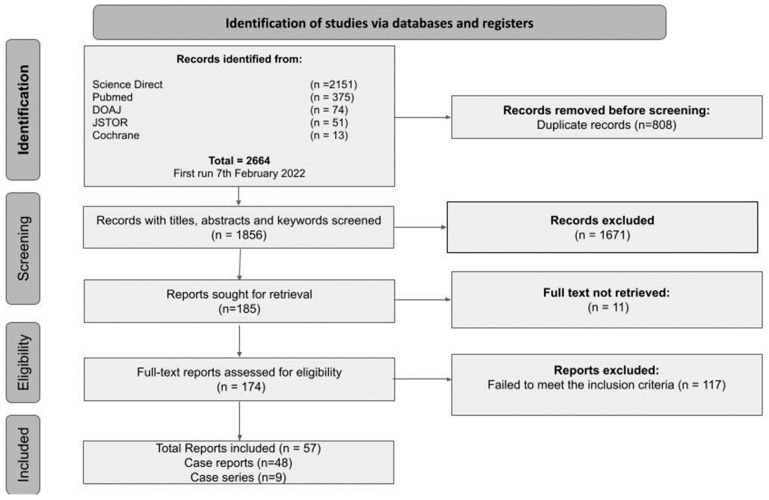
PRISMA flowchart outlining the study search.

In our systematic review, 2664 records were initially identified from the search strategy, of which only 1856 were retrieved after removing 808 duplicate articles. A total of 1671 records were excluded after screening the title and the abstract, giving us a total of 185 records for the full-text screening. Among these, 11 records could not be retrieved, and 117 failed to meet the inclusion criteria and were, therefore, excluded. Finally, a total of 57 articles were included in the systematic review, among which 48 were identified as case reports and 9 as case series (Figure 1).

### 3.2. Patient Characteristics

Overall, we identified a total of 48 case reports and 9 case series, amounting to 118 patients in total. Males were seen to be predominantly affected by BS, as 68 participants were male and 50 were female. The age of diagnosis varied from 22.6 gestational weeks to 59 years, and the majority of patients were less than 5 years of age (66.1%).

Out of the 100 patients assessed for the genetic type of BS, 59 were reported to have Type Ⅲ BS (*n* = 59), followed by Type Ⅱ (*n* = 19), Type I (*n* = 14), Type Ⅳ (*n* = 7), and only 1 case was classified as Type Ⅴ BS (Table 1). Genetic-based testing to confirm the diagnosis of the patients was reported in 31.4% of the patients. Furthermore, consanguinity was observed in about 20% (21/118) of our cases.

**Figure 2:**
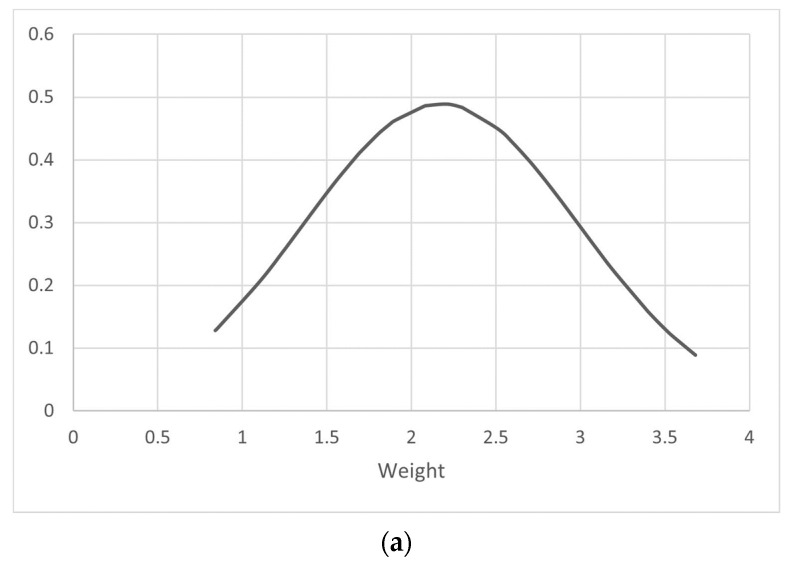

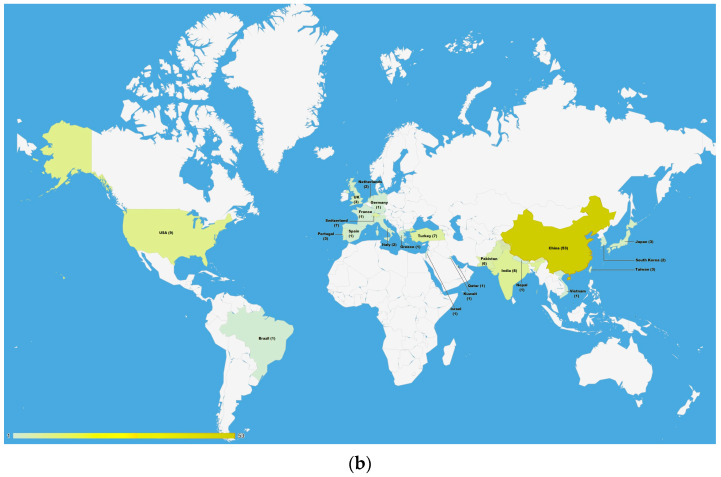
(**a**). The frequency distribution curve of body weight for identified BS patients. (**b**). Geographical distribution of identified cases of BS.

Most of the BS patients were followed up between 1 month and 14 years, with an average follow-up time of 3.35 years. The average birth weight was calculated to be 2.17 ± 0.81, with the lowest birth weight, reported by Azzi et al. [58], being 0.84 kg (Table 2), and the highest being 3.68 kg, reported by Adachi et al. [11] (Table 2). The distribution curve of the measured weights among the reported BS cases is shown in Figure 2a.

### 3.3. Epidemiology/Case Distribution

The geographical spread of BS cases is shown in Figure 2b. Most of the cases were reported in Asia (*n* = 85), followed by Europe (*n* = 23), North America (*n* = 9), and South America (*n* = 1). No cases were reported from Africa, Australia, or Antarctica. The maximum number of cases was seen in the year 2020 (*n* = 55), followed by 2015 (*n* = 12), 2021 (*n* = 11), 2016 (*n* = 9), 2012 (*n* = 8), 2014 (*n* = 8), 2013 (*n* = 6), 2017 (*n* = 5), 2018 (*n* = 3), 2019 (*n* = 1), and 0 in 2022.

### 3.4. Clinical Presentation

A detailed clinical presentation of all of the included cases is described in Supplementary Table S1. More than half (61%) of the patients presented with polyuria, followed by failure to thrive (49.1%), polydipsia (40.7%), nephrocalcinosis (16.1%), dehydration (13.5%), asthenia (11.9%), irritability (3.4%), and 2.5% presented with low birth weight and fever.

Furthermore, 62.7% presented with gastrointestinal symptoms such as vomiting, diarrhea, and constipation. About 15.2% presented with neurological deficits (seizures, hypotonia, hypertonia, and carpopedal spasm), and respiratory distress was found in 7.6% of the patients.

Additionally, 3.4% of the cases were associated with developmental anomalies such as macrocephaly and peculiar facies (e.g., triangular-shaped face, high forehead, asymmetric eyelids, retrognathism, or low-set prominent ears), with 13.5% reporting a premature birth. Furthermore, 33.05% were reportedly affected by polyhydramnios during the antenatal period.

### 3.5. Laboratory Findings

The hypokalemic presentation was seen in the majority of the patients (77.9%), followed by metabolic alkalosis (66.9%), hyperaldosteronism (59.3%), hyperreninemia (58.5%), and hyponatremia (54.2%). Furthermore, hypochloremia was seen in 50% of the patients, followed by hypercalciuria in 12.2%, and hypomagnesemia in 9.3% of the cases.

### 3.6. Management

The treatment options for each case are described in Supplementary Table S1. Most of the cases (74/118) were treated with indomethacin, along with fluids and electrolyte therapy (86/118), which were given intravenously, orally, or as a change in diet plan. Alternatively, a dual therapy with indomethacin and spironolactone was given to 55 patients (46.6%). A total of six patients (5%) received indomethacin therapy via amniocentesis for prenatal management. Furthermore, antiemetics/anticonvulsants/calcimimetics were added as required by the patients.

### 3.7. Quality Assessment

The Supplementary Materials contain figures for the quality assessment of our included studies. For the case reports (Supplementary Figure S1), most of the included studies described the patient characteristics clearly, including the clinical condition on presentation, except for Sobash et al. [47], Verma et al. [50], and Yaqub et al. [55]. The assessment methods and the results were clearly described in all of the reports, except for Alasfour et al. [15] and Raza et al. [45]. Most of the reports gave clear descriptions of the intervention or treatment procedures, along with the post-intervention clinical condition.

For the included case series (Supplementary Figure S2), the quality assessment of the included studies identified two studies [59,61] as high quality, six studies [58,62,63,64,65,66] as medium quality, and one study [60] as low quality. Only five [59,61,62,64,66] studies gave clear criteria for inclusion in the case series. The condition was not measured in a standard, reliable way for the included participants in two studies [60,63]. All of the studies used a valid method for the identification of the condition. There were only two [58,66] studies in which the reporting of the demographics of the participants was not clearly defined, and only one [66] study in which the reporting of the clinical information of the participants was not clear. The outcomes and follow-up results were clearly presented in only four studies [59,61,63,65].

## 4. Discussion

This systematic review aimed to analyze and highlight the various clinical manifestations that a BS patient can display to a clinician. To our knowledge, no previous systematic reviews have been conducted on BS; therefore, ours is the first study on the topic. Most of the patients were diagnosed during the first decade of their lives. The majority of the patients were male and exhibited a positive response to the treatment. Consanguinity was seen in a minority of cases. We found that polyuria was the most reported presentation, while fever was the least common. Most of the patients suffering from BS were found to be male, and most were found to be suffering from Type III BS. We also noted that the majority of patients were diagnosed clinically, and not by genetic-based testing. A near-normal distribution of birth weights was seen across all of the cases. Most of the cases came from China, and the maximum number of cases reported in a year was in 2020. Almost one third of the patients were found to have polyhydramnios, and a tenth were found to be born prematurely. We were also able to identify four cases in which an initial rare hyperkalemic presentation was noted (Table 2). Hypokalemia and metabolic acidosis were reported to be the most common lab findings. The treatment options were mainly limited to indomethacin, and spironolactone, with added supplements as per the requirement of the patient. The results of the study can be utilized in diagnosis and patient management.

The successful management of BS hinges upon early identification, coupled with the expertise of the attending physician. Our systematic review has revealed that only 31% of patients were diagnosed through genetic testing. Considering the high analytical sensitivity of over 90% and clinical sensitivity of approximately 75% in children [2,67,68] and 12.5% in adults, as reported by a recent consensus of experts from Europe [69], genetic testing remains an underutilized resource, likely contributing to the significant frequency of delayed BS detection worldwide. Furthermore, due to the potential overlapping of biochemical markers and clinical symptoms with Gitelman’s syndrome (GS), genetic analysis assumes a crucial significance in ensuring an accurate diagnosis in cases of BS. Even though the overlap between the two might be clinically challenging to differentiate, studies have pointed out some significant differences. BS is notably associated with a more pronounced failure to thrive and growth retardation, compared to GS. Furthermore, individuals with BS often present with hypercalciuria, predisposing them to nephrocalcinosis and nephrolithiasis [70]. It is worth noting that, while the thiazide test has been proposed as a useful diagnostic tool for differentiation, its appropriateness for children under the age of seven remains a subject of debate, due to concerns regarding potential volume depletion [71].

There were three occasions of death (Table 2) reported in our review. A patient in a case by Afzal et al. [12] died due to a sudden cardiopulmonary arrest, leading to instant death, highlighting the unpredictable nature of the CVS complications in BS, where sudden cardiac events can occur, even with prompt medical interventions. This case underscores the need for heightened vigilance in managing electrolyte imbalances in order to prevent such catastrophic events. In another case reported by Akuma et al. [14], the constant deterioration of lung function led to the patient’s demise. In a case by Soumya et al. [48], the patient succumbed to aspiration pneumonia, leading to his eventual death.

Although rare, BS can also be present in adults. We found 10 such cases where patients presented for the first time in adulthood (Table 2), usually with nephrocalcinosis, fatigue, periodic mild paralysis, muscle cramps, and other unusual blood chemistry, as seen in BS. Interestingly, Özdemir et al. [40] discussed the possible etiological relationship between adult BS and mania-like symptoms, where electrolyte disturbances, such as hypokalemia, hyponatremia, and metabolic alkalosis, have been suggested as the possible causes, while others have suggested that mood swings are linked to these imbalances [72,73]. Hatta et al. [74] included some patients presenting with acute psychotic episodes of schizophrenia with hypokalemia.

Even though BS characteristically presents with hypokalemia, certain reports have discussed otherwise. We have identified six such case reports, in which an initial hyperkalemic presentation was seen. Mani et al. [35] suggest that an early postnatal transient hyperkalemia with a history of prematurity, polyuria, and polyhydramnios should raise suspicion for antenatal BS, due to KCNJ1 mutation. Adding to that, a report by Akuma et al. [14] suggests that physicians must be aware of the Type II subtype of neonatal BS, which presents with early transient hyperkalemia, ultimately preventing a misdiagnosis, such as pseudohypoaldosteronism or otherwise.

Under stressful situations, such as surgical procedures, the body fluid levels may rapidly change, and pose a significant challenge for both anesthesiologists and surgeons. Raza and colleagues [45] talk about such a case, emphasizing that the management of these patients requires a special focus on the maintenance of cardiovascular stability, control of the plasma potassium level, and the prevention of renal damage. This adds to the already established guidelines on the perioperative management of patients with inherited salt-wasting alkalosis. These guidelines [75] highlight the importance of preoperative risk assessment, considering factors such as the nature of the surgery, concurrent medications, and cardiovascular risk factors. Additionally, they highlight the significance of electrolyte stability and the avoidance of rapid preoperative correction. The guidelines [75] set the minimum acceptable potassium levels (3.0 mmol L^−1^) based on the serum magnesium levels (≥0.5 mmol L^1^) and suggest appropriate monitoring during anesthesia and recovery.

Those born from consanguineous marriages have a greater probability of inheriting defective recessive genes [76]. Autosomal-recessive disorders like BS have been reported widely in communities with high consanguinity rates [77]. It has been widely established in the clinical literature that such marriages lead to an increased expression of autosomal-recessive disorders, increased birth defects, and mortality in offspring, as reported by many other studies [76,78,79,80]. Our review has revealed a comparable trend, with 21 out of 118 cases of BS being reported in the offspring of consanguineous couples.

## 5. Limitations and Strengths

To the best of our knowledge, our study is the first systematic review summarizing the available clinical literature in the form of case reports and case series on BS. We present a comprehensive overview of the published data, with a robust quality appraisal of the included studies.

We acknowledge that this systematic review had its limitations. We only included case reports and case series, due to the limited literature published on BS; therefore, there is a potential risk of bias. After a thorough screening and data collection, we were not able to retrieve all of the required information in all of the categories. These missing data were associated with some skewness among the datasets. Some of the patients were diagnosed based on clinical signs and symptoms and not actual genetic testing. Since we only included papers from the last 10 years reporting BS, we might have missed important clinical data from prior years. In addition, it is noteworthy that not all of the patients received genetic diagnoses, as some were clinically diagnosed. However, it is essential to underscore our rigorous review process, wherein each case report or series underwent a thorough examination by at least two independent authors. Only the papers in which the attending physician conclusively diagnosed BS were considered for inclusion. Nevertheless, while we maintain a high degree of confidence in our diagnostic criteria, we acknowledge that a very minimal proportion could potentially represent GS rather than BS.

## 6. Conclusions

Although Bartter syndrome is a rare diagnosis, we were able to summarize the clinical characteristics, presentation, and treatment from all five types reported through a robust systematic review, including the literature from the past decade. For BS testing, premature neonates with unexplained polyhydramnios, growth retardation, or electrolyte abnormalities should be investigated. The clinical presentation, epidemiology, treatment options, and follow-up of the BS patients presented in this review could be useful for physicians in clinical practice.

## Figures and Tables

**Table 1:** Laboratory analysis and period of follow-up of the included studies in our systematic review.

First Author	Study Design	Number of Cases	Bartter Syndrome Type	Laboratory Evaluation					Period of Follow-Up
				Hypokalemia	Hyponatremia	Hyperreninemia	Metabolic Alkalosis	Hyperaldosteronism	
**Abdelgadir et al.** [10]	CR	1	Type Ⅲ	Y	Y		Y		
**Adachi et al.** [11]	CR	1	Type III	Y	-	Y	Y	Y	14 yr
**Afzal et al.** [12]	CR	1	Type I	Y	Y	Y	Y	Y	
**Agrawal et al.** [13]	CR	1	Type III	Y	Y		Y		1 mth
**Akuma et al.** [14]	CR	1	Type Ⅱ		Y	N		Y	
**Alasfour et al.** [15]	CR	1	Type I	Y	Y	N	Y		
**Alhammadi et al.** [16]	CR	1	Type I	Y			Y		
**Chiang et al.** [17]	CR	1	Type Ⅲ	Y		Y	Y		
**Cho et al.** [18]	CR	1	Type III	Y	Y				
**Chuang et al.** [19]	CR	1	Type I	Y			Y		
**Coroado et al.** [20]	CR	1	Type Ⅳ						7 mth
**Cruz et al.** [21]	CR	1	Type Ⅲ	Y	N	Y	Y	Y	2 yr
**Fretzayas et al.** [22]	CR	1	Type Ⅱ	Y	Y	Y	Y	Y	3 yr
**Gargano et al.** [23]	CR	1	Type III	Y	Y	-	Y		1 yr
**Gollasch et al.** [24]	CR	1	Type Ⅱ	Y		Y		Y	3 yr
**Gross et al.** [25]	CR	1	Type I	N	N		N		10 mth
**Hegde et al.** [26]	CR	1	Type I	Y	Y	Y	Y		
**Heilberg et al.** [27]	CR	1	Type Ⅳ	Y		Y		Y	2 w
**Huang et al.** [28]	CR	1	Type Ⅱ	Y	N	Y	Y	Y	1 yr
**Hussain et al.** [29]	CR	1	Type Ⅴ	Y	Y		Y		3 mth
**Khan et al.** [30]	CR	1		Y		Y		Y	
**Khandelwal et al.** [31]	CR	1	Type Ⅱ	N	N	N	N	N	
**Le et al.** [32]	CR	1	Type Ⅲ	Y	Y	Y	Y		
**Li et al.** [33]	CR	1	Type I						
**Mali et al.** [34]	CR	1	Type Ⅲ	Y	Y		Y	Y	
**Mani et al.** [35]	CR	1	Type Ⅱ	N	Y	Y	Y	Y	5 mth
**Maruyama et al.** [36]	CR	1	Type I		Y	Y		Y	7 mth
**Mou et al.** [37]	CR	1	Type Ⅲ	Y		Y	Y		2.9 yr
**Nam et al.** [38]	CR	1	Type I	Y	Y	Y	N	Y	3 mth
**Ozdemir et al.** [39]	CR	1		Y	Y	Y	Y	Y	
**Özdemir et al.** [40]	CR	1	-	Y	Y	-	Y	-	
**Pablos et al.** [41]	CR	1	Type Ⅳ	Y	Y	Y		Y	2 yr
**Plumb et al.** [42]	CR	1	Type Ⅳ	Y			Y		1 yr
**Preshaw et al.** [43]	CR	1	Type I		Y				2 yr
**Rachid et al.** [44]	CR	1	Type I						
**Raza et al.** [45]	CR	1		Y					2 mth
**Sakallı et al.** [46]	CR	1	Type Ⅳ	Y	Y	Y	Y	Y	16 mth
**Sobash et al.** [47]	CR	1		Y					1 mth
**Soumya et al.** [48]	CR	1		Y	Y	Y	Y		
**Vergine et al.** [49]	CR	1	Type I					Y	1.5 yr
**Verma et al.** [50]	CR	1		Y		Y	Y	Y	3 mth
**Vieira et al.** [51]	CR	1	Type Ⅲ	Y	Y	Y	Y	Y	1 yr
**Wang et al.** [52]	CR	1	Type Ⅳ					Y	2 yr
**Westland et al.** [53]	CR	1	Type Ⅲ	Y	Y		Y		-
**Wu et al.** [54]	CR	1	Type III	Y		Y		Y	10 yr
**Yaqub et al.** [55]	CR	1		Y		Y	Y	Y	1 mth
**Yoshioka et al.** [56]	CR	1		Y	Y		Y	Y	12 d
**Zhu et al.** [57]	CR	1	Type III	Y		Y	Y	Y	14 yr
**Azzi et al.** [58]	CS	1/7	Type Ⅲ	Y					4 w
		2/7	Type Ⅳ		Y				4 w
		3/7	Type I						4 w
		4/7							4 w
		5/7	Type Ⅱ						4 w
		6/7	Type Ⅱ						4 w
		7/7	Type I						4 w
**Buyukcelik et al.** [59]	CS	1/3		Y	Y	Y	Y	Y	12 yr
		2/3		Y	Y	Y	Y	Y	2 yr
		3/3		Y	Y	Y	Y	Y	11 yr
**Çetinkaya et al.** [60]	CS	1/2		Y	Y	Y	Y	Y	-
		2/2					Y		-
**Han et al.** [61]	CS	42	Type Ⅲ	Y (39/42)	Y (27/42)	Y (33/35)	Y (39/42)	Y (33/35)	21 mth 9–38 mth (36/42)
**Hussain et al.** [62]	CS	1/2		Y	Y	Y	Y	Y	9 mth
		2/2		Y	Y	Y	Y	Y	2 mth
**London et al.** [63]	CS	1/5(V-8)	Type Ⅱ		Y	Y		Y	7 yr
		2/5(V-10)	Type Ⅱ	Y					20 yr
		3/5(V-11)	Type Ⅱ	Y		Y		Y	28 yr
		4/5(V-3)	Type Ⅱ						7 yr
		5/5(V-4)	Type Ⅱ	Y	Y			Y	9 yr
**Sharma et al.** [64]	CS	1/2	Type Ⅱ	Y	Y	Y	Y	Y	-
		2/2	Type I	Y	Y	Y	Y	Y	-
**Yang et al.** [65]	CS	1/2	Type Ⅲ	Y	Y	Y	Y	Y	6 yr
		2/2	Type Ⅲ	Y	Y	Y	Y	Y	2 yr
**Zuo et al.** [66]	CS	1/5	Type Ⅱ	Y					2.58 yr
		2/5	Type Ⅱ	N					3.5 yr
		3/5	Type Ⅱ	Y					0.5 yr
		4/5	Type Ⅱ	N					1 yr
		5/5	Type Ⅱ	N					1.83 yr

d, Day(s); w, week(s); mth, month(s); yr, year(s); Y, Yes; N, No; CR, Case Report; CS, Case Series.

**Table 2:** Patient demographic and other clinical characteristics.

First Author	Number of Cases	Country	Gender	Adult First Time Bs Presentation	Age of Diagnosis	Birth Weight	Bs Type	Consanguineous Couple (Y/N)	Initial Hyperkalemic Presentation	Mortality (Y)
Abdelgadir et al. [10]	1	Qatar	F		20 d	3.09 Kg	Type Ⅲ	Y		
Adachi et al. [11]	1	Japan	M		8 mth	3.68 Kg	Type III			
Afzal et al. [12]	1	Pakistan	F		At birth	1.4 Kg	Type I	Y		Y
Agrawal et al. [13]	1	Nepal	F		14 mth		Type III	N		
Akuma et al. [14]	1	UK	M			1.33 Kg	Type Ⅱ	Y	Y	Y
Alasfour et al. [15]	1	Kuwait	M		5 mth	1.7 Kg	Type I			
Alhammadi et al. [16]		Qatar					Type I			
Chiang et al. [17]	1	Taiwan	F	Y	45 yr		Type Ⅲ			
Cho et al. [18]	1	Republic of Korea	M		10 yr	3.5 Kg	Type III			
Chuang et al. [19]	1	Taiwan	M		5 yr		Type I			
Coroado et al. [20]	1	Portugal	M		30 WOG	2.88 Kg	Type Ⅳ	N		
Cruz et al. [21]	1	Portugal	F	Y	32 yr		Type Ⅲ			
Fretzayas et al. [22]	1	Greece	M			1.41 Kg	Type Ⅱ	N	Y	
Gargano et al. [23]	1	Italy	F		3 mth	2.9 Kg	Type III	Y		
Gollasch et al. [24]	1	Germany	F	Y	43 yr		Type Ⅱ			
Gross et al. [25]	1	Israel	M		5 mth	1.1 Kg	Type I	Y		
Hegde et al. [26]	1	India	F			0.85 Kg	Type I	Y		
Heilberg et al. [27]	1	Brazil	M	Y	20 yr		Type Ⅳ	Y		
Huang et al. [28]	1	The Netherlands	M	Y	35 yr		Type Ⅱ	N		
Hussain et al. [29]	1	India	F	Y	59 yr		Type Ⅴ			
Khan et al. [30]	1	Pakistan	F	Y	28 yr					
Khandelwal et al. [31]	1	India	F		14 yr	2.75 Kg	Type Ⅱ	N		
Le et al. [32]	1	Vietnam	M		13 yr		Type Ⅲ	N		
Li et al. [33]	1	USA	M		12 yr	2 Kg	Type I	N		
Mali et al. [34]	1	India	F		5 yr		Type Ⅲ	N		
Mani et al. [35]	1	USA	M			1.56 Kg	Type Ⅱ	N	Y	
Maruyama et al. [36]	1	Japan	M			1.34 Kg	Type I	N		
Mou et al. [37]	1	China	M	Y	48 yr		Type Ⅲ	Y		
Nam et al. [38]	1	Republic of Korea	M		31 WOG	2.21 Kg	Type I			
Ozdemir et al. [39]	1	Turkey	M		28 WOG	1.24 Kg		N		
Özdemir et al. [40]	1	USA	M		33 yr		-			
Pablos et al. [41]	1	Spain	F			2.08 Kg	Type Ⅳ	N		
Plumb et al. [42]	1	UK	F		2 w	1·68 Kg	Type Ⅳ	Y		
Preshaw et al. [43]	1	UK	M			1.9 Kg	Type I			
Rachid et al. [44]	1	France	F				Type I	Y		
Raza et al. [45]	1	Pakistan	M							
Sakallı et al. [46]	1	Turkey	M		8 mth	1.8 Kg	Type Ⅳ	Y		
Sobash et al. [47]	1	USA	F		47 yr					
Soumya et al. [48]	1	India	M		4 mth	2.3 Kg		N		Y
Vergine et al. [49]	1	Italy	M		1 yr	1.2 Kg	Type I			
Verma et al. [50]	1	India	M		10 yr					
Vieira et al. [51]	1	Portugal	F		11 mth	2.51 Kg	Type Ⅲ	N		
Wang et al. [52]	1	China	F			1.45 Kg	Type Ⅳ	N		
Westland et al. [53]	1	The Netherlands	F		35 WOG	3.4 Kg	Type Ⅲ	N		
Wu et al. [54]	1	China	F		5 mth	3.2 Kg	Type III			
Yaqub et al. [55]	1	Pakistan	M	Y	38 yr					
Yoshioka et al. [56]	1	Japan	M		53 yr					
Zhu et al. [57]	1	China	F		15 yr	2.3 Kg	Type III	N		
Azzi et al. [58]	1/7	Switzerland	F			1.45 Kg	Type Ⅲ	Y		
	2/7		F			0.84 Kg	Type Ⅳ	Y		
	3/7		F			1.43 Kg	Type I	Y		
	4/7		M			2.75 Kg		N		
	5/7		F			1.47 Kg	Type Ⅱ	N		
	6/7		M			2.3 Kg	Type Ⅱ	N		
	7/7		M			1.37 Kg	Type I	N		
Buyukcelik et al. [59]	1/3	Turkey	M		11 yr			N		
	2/3		M		11 yr			N		
	3/3		F		10 yr			N		
Çetinkaya et al. [60]	1/2	Turkey	F		31 WOG	3.5 Kg				
	2/2		M		37 WOG	3.4 Kg		N	Y	
Han et al. [61]	42	China	26 M 16 F		37 ± 31 mth		Type Ⅲ	1/42		
Hussain et al. [62]	1/2	Pakistan	M		20 d			Y		
	2/2		M		2 mth			Y		
London et al. [63]	1/5 (V-8)	USA	M		2.3 yr	1.8 Kg	Type Ⅱ	Y	Y	
	2/5 (V-10)		F		11 yr	2.7 Kg	Type Ⅱ	Y		
	3/5 (V-11)		F		4 yr	1.4 Kg	Type Ⅱ	Y		
	4/5 (V-3)		F		5 yr	2.8 Kg	Type Ⅱ	Y		
	5/5 (V-4)		F			2.6 Kg	Type Ⅱ		Y	
Sharma et al. [64]	1/2	India	M		35 WOG	1.8 Kg	Type Ⅱ	N		
	2/2		M		9 ½ mth	3 Kg	Type I	N		
Yang et al. [65]	1/2	China	F		4 mth	2.9 Kg	Type Ⅲ	N		
	2/2		M		4.1 yr	3.4 Kg	Type Ⅲ	Y		
Zuo et al. [66]	1/5	China	M	Y	25 yr		Type Ⅱ	N		
	2/5		M		7 yr		Type Ⅱ	N		
	3/5		F		5.8 yr		Type Ⅱ	N		
	4/5		M		3.1 yr		Type Ⅱ	N		
	5/5		F		1.8 yr		Type Ⅱ	N		

CR, Case Report; CS, Case Series; M, Male; F, Female; Y, Yes; N, No; d, Day(s); w, week(s); mth, Month(s); yr, Year(s); WOG, weeks of gestation.

## Data Availability

The data presented in this study are available within the article and its Supplementary Materials.

## Supplementary Materials

The following supporting information can be downloaded at: https://www.mdpi.com/article/10.3390/medicina59091638/s1, Figure S1: Quality Assessment of Included Case Reports based on JBI (Joanna Briggs Institute) Critical Appraisal Checklist for Case Reports; Figure S2: Quality Assessment of Included Case Series Based on JBI (Joanna Briggs Institute) Critical Appraisal Checklist for Case Series; Table S1: Detailed Treatment options and First Presentations for Bartter Syndrome Patients.
