# Supplementary material for: Bartter Syndrome: A Systematic Review of Case Reports and Case Series
Source: Medicina (Kaunas). 2023 Sep 11;59(9):1638. doi: 10.3390/medicina59091638 (PMC10537044; doi:10.3390/medicina59091638)
Supplement: Supplementary file 1 [file medicina-59-01638-s001.zip › medicina-2573589-supplementary.pdf]

Supplementary Figure S1: Quality Assessment of Included Case Reports based on JBI (Joanna Briggs Institute) Critical Appraisal Checklist for Case Reports.

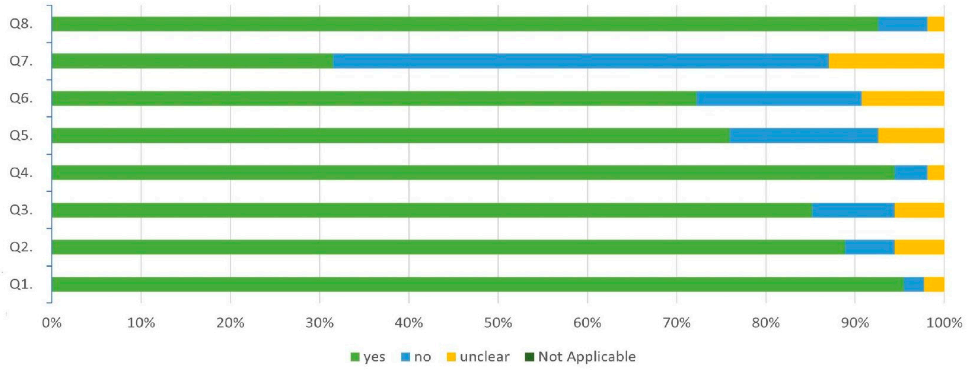

	Q1. Were patient's demographic characteristics clearly described?	Q2. Was the patient's history clearly described and presented as a timeline?	Q3. Was the current clinical condition of the patient on presentation clearly described?	Q4. Were diagnostic tests or assessment methods and the results clearly described?	Q5. Was the intervention(s) or treatment procedure(s) clearly described?	Q6. Was the post-intervention clinical condition clearly described?	Q7. Were adverse events (harms) or unanticipated events identified and described?	Q8. Does the case report provide takeaway lessons?
yes	42	48	46	51	41	39	17	50
no	1	3	5	2	9	10	30	3
unclear	1	3	3	1	4	5	7	1
Not Applicable								

yes no unclear Not Applicable

Supplementary Figure S2: Quality Assessment of Included Case Series Based on JBI (Joanna Briggs Institute) Critical Appraisal Checklist for Case Series.

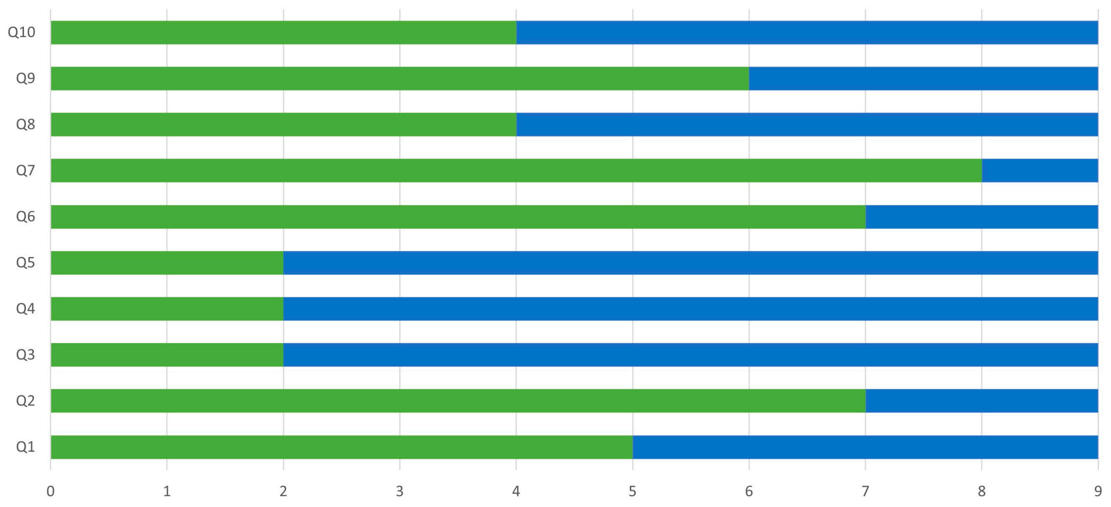

	Q1. Were there clear criteria for inclusion in the case series?	Q2. Was the condition measured in a standard, reliable way for all participants included in the case series ?	Q3. Were valid methods used for identification of the condition for all participants included in the case series?	Q4. Did the case series have consecutive inclusion of participants?	Q5. Did the case series have complete inclusion of participants?	Q6. Was there clear reporting of the demographics of the participants in the study?	Q7. Was there clear reporting of clinical information of the participants?	Q8. Were the outcomes or follow up results of cases clearly reported?	Q9. Was there clear reporting of the presenting site(s)/clinic(s) demographic information?	Q10. Was statistical analysis appropriate?
yes	5	7	2	2	2	7	8	4	6	4
no	4	2	7	7	7	2	1	5	3	5
unclear										
Not Applicable										

Supplementary Table S1: Detailed Treatment options and First Presentations for Bartter Syndrome Patients.

Serial No.	Ref.	Study Design	Number of cases	Treatment	Patient's first presentation
1	Abdelgadir et al. [10]	Case Report	1	Oral potassium chloride, sodium chloride, indomethacin, and spironolactone	Persistent non-bilious vomiting, moderate dehydration, constipation, and mild abdominal distension
2	Adachi et al. [11]	Case Report	1	Potassium (K), sodium chloride, spironolactone, and anti-inflammatory agents	Failure to thrive and mild intellectual impairment
3	Afzal et al. [12]	Case Report	1	Indomethacin	Dehydration, polyuria, and unable to tolerate oral feed
4	Agrawal et al. [13]	Case Report	1	Indomethacin and supportive management for failure to thrive	Child looked sick and irritable, unable to gain weight, and recurrent episodes of vomiting
5	Akuma et al. [14]	Case Report	1	Sodium supplementation, fluid replacement, and calcium and magnesium supplementation	Polyuria
6	Alasfour et al. [15]	Case Report	1	Spironolactone, oral potassium chloride, and sodium chloride	

7	Alhammadi et al. [16]	Case Report			Intermittent non-bilious vomiting
8	Chiang et al. [17]	Case Report	1	Spironolactone, potassium chloride supplementation, and high K+ diet.	Muscle strength and reflexes over the bilateral lower extremities were diminished, chronic severe hypokalemia, and episodes of muscle paralysis
9	Cho et al. [18]	Case Report	1	Indomethacin	Chronic persistent hypokalemia, hypomagnesemia, and acute heart failure
10	Chuang et al. [19]	Case Report	1	Oral potassium chloride and spironolactone	Polyuria, Polydipsia, vomiting, and asthenia
11	Coroado et al. [20]	Case Report	1	Indomethacin and potassium chloride	Macrocephaly and peculiar facies, axial hypotonia with peripheric hypertonia and abnormal posturing of the extremities, neurosensorial deafness, polyuria, polydipsia and failure to thrive, born prematurely due to severe polyhydramnios
12	Cruz et al. [21]	Case Report	1	Oral potassium chloride supplements, 4800 mg/day in four divided doses; oral magnesium aspartate supplements, 1229.6mg three times daily; spironolactone, titrated to a maximal dose of 300 mg/day; and low dose of ramipril, 1.25 mg/day	Asthenia, polyuria, nocturia, carpopedal spasms, and persistent hypokalemia
13	Fretzayas et al. [22]	Case Report	1	Indomethacin (2 mg/Kg/d) and hydrochlorothiazide (1 mg/Kg/d)	Polyuria and severe weight loss
14	Gargano et al. [23]	Case Report	1	IV lorazepam and phenobarbital used to stop seizures; intravenous bolus of 60 mL of 0.9% sodium chloride because of the severe dehydration, and, following adequate intravascular volume expansion, rehydration was achieved with appropriate fluids; indomethacin	Diarrhea, severe dehydration, malnourished, numbness, hypotonia, hyporeactivity, generalized seizures with tonic flexion of the upper limbs, tonic extension of the lower limbs and right eye deviation
15	Gollasch et al. [24]	Case Report	1	Oral supplementation of potassium (80 mmol/day) and ramipril (10 mg/day)	Bilateral nephrocalcinosis
16	Gross et al. [25]	Case Report	1	Intravenous fluids and oral potassium citrate	Severe diarrhea, failure to thrive, nephrocalcinosis, drowsiness, and hypotonia.
17	Hegde et al. [26]	Case Report	1	Indomethacin was started at 0.1 mg/Kg in 12 hourly intravenous injections, but the dose had to be gradually increased to 0.25 mg/Kg/dose 12 hourly by day 9, and spironolactone	Tachycardia, dehydration, and polyuria
18	Heilberg et al. [27]	Case Report	1	Intravenous potassium chloride (KCI) replacement was started with 19.1% 20mL, followed by oral KCI supplementation with 6.0% 20mL t.i.d. Two weeks after hospital discharge, spironolactone (100mg/day) was added to the oral KCI supplementation (30mEq/day)	Asthenia
19	Huang et al. [28]	Case Report	1	Oral potassium and oral spironolactone	Lower back pain

20	Hussain et al. [29]	Case Report	1	Patient was put on potassium, calcium, and magnesium supplements in hospital and patient improved. Patient was discharged on diuretics (torsemide/spironolactone combination), ramipril, calcium, and magnesium supplements	Episode of seizure, easy fatigability, asthenia, and bilateral upper lobe fibrosis of lungs
21	Khan et al. [30]	Case Report	1	Potassium chloride, calcium, and vitamin D	Asthenia, fatigue, and severe left hip pain.
22	Khandelwal et al. [31]	Case Report	1	Potassium citrate supplements and hydrochlorothiazide	Polydipsia, polyuria, and abdominal pain
23	Le et al. [32]	Case Report	1	Enalapril 0.5mg/Kg/day	Syncope and hypertension
24	Li et al. [33]	Case Report	1	Cinacalcet and potassium citrate, with subsequent reduction in PTH, and serum calcium	Premature birth at 33.5 weeks gestation.
25	Mali et al. [34]	Case Report	1	IV normal saline along with potassium, antiemetics, and H2 blockers, indomethacin, and aldosterone antagonist	Vomiting and dehydration
26	Mani et al. [35]	Case Report	1	The infant received supportive care. Fluid and electrolyte supplements for the first 2 weeks. Enteral sodium supplements (oral sodium chloride solution 4 mEq/mL) on DOL 13. On DOL 24, enteral potassium supplements (oral potassium chloride solution 20 mEq/15mL). By DOL 28, the infant had been weaned off both the supplements	Respiratory distress syndrome, polyuria, and bilateral pyelectasis in the fetus
27	Maruyama et al. [36]	Case Report	1	Calcium IV 600mg/Kg/d from day 0, calcium lactate at day 19, vitamin D (0.05 µg/Kg/d) at day 20	Polyuria
28	Mou et al. [37]	Case Report	1	Potassium chloride (KCl) (2.0 g/day), spironolactone (40 mg/day), and potassium magnesium aspartate (8 tablets/day) were prescribed to II-2 to correct electrolytic disturbances. Benzbromarone (50 mg/day) was prescribed to correct hyperuricemia. The arthralgia persisted. Febuxostat (50 mg/day) was, therefore, added to further lower UA	Fatigue, intermittent carpopedal spasm, paroxysmal fatigue, salting craving, polydipsia, polyuria, episodic carpopedal spasm, intense pain, swelling in the proximal interphalangeal (PIP) joint of the right index finger, and bilateral knees and heels were also involved later
29	Nam et al. [38]	Case Report	1	Antenatal indomethacin therapy	Low birth weight and polyuria
30	Ozdemir et al. [39]	Case Report	1	Endotracheal surfactant, standard fluid regimen, antibiotics, total parenteral nutrition (TPN), including SMOF lipid 20% and primene 10%; f/b fluid correction with total maintenance fluid >250 mL/Kg/day (DOL6); indomethacin 1 mg/Kg (postnatal wk 21)	Breathing was absent at birth; respiratory distress symptoms, including tachypnea, nasal flaring, grunting, and retractions
31	Özdemir et al. [40]	Case Report	1		Psychiatric examination shows decreased self-care; open conscious and normal orientation; answering appropriately to questions; visual hallucination; decreased concentration and attention; ideas of reference; grandiosity; illogical thinking; affective instability; dysphoric mood; irritability; increased psychomotor activity; aggressive behavior; having no insight. Decreased appetite, sleep

					restlessness, aggression, agitation, rapidly fluctuating emotions, talking to oneself, suspicion, and mistrust of the people.
32	Pablos et al. [41]	Case Report	1	Fluid intake of 300 mL/Kg/day, up to 15 mmol/Kg/day of NaCl, 4 mmol/Kg/day of KCl supplements, respectively, and indomethacin (2.5 mg/Kg/day, 3 doses)	Severe polyuria, dehydration, and axial hypotonia
33	Plumb et al. [42]	Case Report	1	Supplementation with sodium (14 mmol/Kg per day), potassium (13 mmol/Kg per day), and fluid (200 mL/Kg per day)	Polyuria, extreme acid–base and electrolyte disturbance, and chronic kidney disease
34	Preshaw et al. [43]	Case Report	1		Polyuria
35	Rachid et al. [44]	Case Report	1		
36	Raza et al. [45]	Case Report	1	Oral potassium supplements, indomethacin 1.5ml 8 hourly, and vitamin D supplements	Respiratory distress, irritable, and malnourished
37	Sakallı et al. [46]	Case Report	1	Indomethacin treatment was initiated at a dose of 1 mg/Kg per day, and the child was maintained on supportive care, potassium supplements, and dietary advice	Dehydrated, facial dysmorphism, non-bilious vomiting, frequent diarrhea episodes, abdominal distension, failure to thrive, hearing loss, and polyuria
38	Sobash et al. [47]	Case Report	1	Magnesium oxide and potassium chloride	Restless leg syndrome, peptic ulcer disease, achalasia, bleeding peptic ulcer, small bowel obstruction, intractable abdominal pain, nausea, loss of appetite, hematemesis, and constipation.
39	Soumya et al. [48]	Case Report	1	Inj. Ceftriaxone, intravenous bolus of both hypertonic saline and potassium chloride, potassium supplements, and intravenous indomethacin	Asthenia, poor feeding, low-grade fever, and abnormal staring for two to three days prior to admission
40	Vergine et al. [49]	Case Report	1		Severe dehydration, intermittent high fever, recurrent vomiting, polyuria, extreme irritability, spasmodic need of water, severe growth delay, with both weight and height below third percentile, and presence of a triangular facies with frontal bossing
41	Verma et al. [50]	Case Report	1	Potassium replacement was carried out using intravenous potassium chloride infusion in mannitol initially, followed by oral replacement	Polyuria, polydipsia, followed by generalized tonic–clonic seizures, and quadriparesis without bladder and bowel involvement, with normal cognition and short stature (height below the third percentile of corresponding age)
42	Vieira et al. [51]	Case Report	1	Potassium chloride and indomethacin	Failure to thrive, mild delay of psychomotor development, polyuria, and polydipsia
43	Wang et al. [52]	Case Report	1		Severe dehydration, polyuria, diarrhea, growth retardation, dysgnosia, profound hearing loss, and bilateral renal pyramid calcification

44	Westland et al. [53]	Case Report	1	At 30 weeks of gestation: Indomethacin and corticosteroids<br>NICU (at birth); NaCl (8.8 mmol/Kg/d), KCl (2.9 mmol/Kg/d), and IV fluids started immediately after birth; indomethacin, gradually increasing in dose from 0.2 mg/Kg/24 h<br>(DOL1) to 2.8 mg/Kg/24 h (DOL8), and spironolactone (5.9 mg/Kg/d) started; Amoxicillin-clavulanic acid (25 mg/Kg/d); intravenous penicillin and amikacin (DOL25)	Congenital anomalies of the kidney and urinary tract (CAKUT) on Prenatal USG
45	Wu et al. [54]	Case Report	1	Oral potassium chloride (4–6 mmol/Kg per day), indomethacin enteric-coated tablets (1–2 mg/Kg per day), and Antiseton (1–3 mg/Kg per day)	Growth retardation, slightly low muscle tension in the lower limbs, aggravated nausea, vomiting accompanied by malnutrition, and mild to moderate dehydration
46	Yaqub et al. [55]	Case Report	1	Potassium-rich diet, potassium replacement, and spironolactone 50 mg twice a day	Fatigue, asthenia and lower limb weakness, bilateral flank pain, and polyuria
47	Yoshioka et al. [56]	Case Report	1	Potassium chloride was given intravenously along with isotonic saline (0.9% sodium chloride + 40 mEq potassium chloride/L) for 24 hours, 1800 mg of potassium chloride was given orally, 50mg of spironolactone, together with 3600 mg of potassium chloride daily	Fatigue, disorientation, fever, and a stinging/tingling sensation on the head
48	Zhu et al. [57]	Case Report	1	Oral spironolactone, indomethacin, and potassium supplements	Frequent vomiting and dehydration
49	Azzi et al. [58]	Case series	Case 1 of 7	During the first 4 weeks, the median supplementations were 163 to 810 mL/Kg/day for fluids, 6.2 to 72 mmol/Kg/ day for sodium, and 118 to 156 Kcal/Kg/day for calories	Antenatal polyhydramnios
			Case 2 of 7	During the first 4 weeks, the median supplementations were 163 to 810 mL/Kg/day for fluids, 6.2 to 72 mmol/Kg/ day for sodium, and 118 to 156 Kcal/Kg/day for calories	Antenatal polyhydramnios
			Case 3 of 7	In utero, indomethacin was initiated between 25 and 27 weeks of gestation. During the first 4 weeks, the median supplementations were 163 to 810 mL/Kg/day for fluids, 6.2 to 72 mmol/Kg/ day for sodium, and 118 to 156 Kcal/Kg/day for calories	Antenatal polyhydramnios
			Case 4 of 7	In utero, indomethacin was initiated between 25 and 27 weeks of gestation. During the first 4 weeks, the median supplementations were 163 to 810 mL/Kg/day for fluids, 6.2 to 72 mmol/Kg/ day for sodium, and 118 to 156 Kcal/Kg/day for calories	Antenatal polyhydramnios
			Case 5 of 7	In utero, indomethacin was initiated between 25 and 27 weeks of gestation. During the first 4 weeks, the median supplementations were 163 to 810 mL/Kg/day for fluids, 6.2 to 72 mmol/Kg/	Antenatal polyhydramnios

				day for sodium, and 118 to 156 Kcal/Kg/day for calories	
			Case 6 of 7	In utero, indomethacin was initiated between 25 and 27 weeks of gestation. During the first 4 weeks, the median supplementations were 163 to 810 mL/Kg/day for fluids, 6.2 to 72 mmol/Kg/day for sodium, and 118 to 156 Kcal/Kg/day for calories	Antenatal polyhydramnios
			Case 7 of 7	In utero, indomethacin was initiated between 25 and 27 weeks of gestation. During the first 4 weeks, the median supplementations were 163 to 810 mL/Kg/day for fluids, 6.2 to 72 mmol/Kg/day for sodium, and 118 to 156 Kcal/Kg/day for calories	Antenatal polyhydramnios
50	Buyukcelik et al. [59]	Case series	Case 1 of 3		Polyuria, restlessness, dehydration, loss of weight gain, and failure to thrive.
			Case 2 of 3		Growth retardation
			Case 3 of 3		Polydipsia, dehydration, constipation, and failure to thrive
51	Çetinkaya et al. [60]	Case series	Case 1 of 2	Oral indomethacin therapy was started at a dose of 1 mg/Kg/d	Severe respiratory distress, cyanosis, intercostal/subcostal retractions, and generalized edema and bilateral pleural effusion
			Case 2 of 2	After 8 weeks of dialysis oral antibiotic prophylaxis	
52	Han et al. [61]	Case series	42 Cases	Potassium chloride (36/36); indomethacin (1.30 mg/Kg [range 0.7–2.4 mg/Kg]) {32/36}; spironolactone (0.96 mg/Kg [range 0.29–2.56 mg/Kg]) {20/32 receiving indomethacin}; magnesium chloride supplementation (4/36)	Slow increase in body weight and growth retardation (23/36)
53	Hussain et al. [62]	Case series	Case 1 of 2	Correction of electrolytes and dehydration, along with indomethacin	Ill-looking, fever, dehydration, and respiratory distress
			Case 2 of 2	Correction of electrolyte imbalance and dehydration, along with oral potassium supplements and indomethacin	Dehydration, asthenia, and failure to gain weight
54	London et al. [63]	Case series	Case 1 of 5 (Proband V-8)	Intravenous calcium gluconate was administered, followed by oral calcium and vitamin D3 supplements. The parents were instructed to discontinue cola beverages, provide the child with a balanced dairy-rich diet, and expose him to sunlight. He was discharged with daily supplements of 500 mg oral calcium and 2000 U of vitamin D3	Generalized seizures, fever of 39°C, hypocalcemia, hyponatremia, hyperkalemia, and concomitant with elevated renin and aldosterone
			Case 2 of 5 (Patient V-10)	Oral calcium, vitamin D, and potassium supplements	Tetany, nephrolithiasis, hydronephrosis, and osteopenia of the wrist

			Case 3 of 5 (Patient V-11)	Oral calcium, Alpha D3, and potassium supplementation	Limping, rickets, premature birth, failure to thrive, polyuria, and polydipsia
			Case 4 of 5 (Patient V-3)		Developmental delay and recurrent vomiting
			Case 5 of 5 (Patient V-4)	Sodium and potassium supplements and indomethacin	Hypotonia, dysmorphic features, and polyuria
55	Sharma et al. [64]	Case series	Case 1 of 2		
			Case 2 of 2		Dehydration
56	Yang et al. [65]	Case series	Case 1 of 2	DOL 2: Spironolactone (1 mg/Kg/d), catopril (1 mg/Kg/d) for oral and adequate intravenous fluid therapy were given;<br>DOL 6: Intravenous therapy was replaced by oral KCl solution (10 mmol/Kg/d);<br>Day 11: The baby was dismissed from hospital in-patient care with the therapy of KCl and increased fluid intake with age	Dehydration, vomiting, delayed development, and nephrocalcinosis
			Case 2 of 2	DOL 1: Spironolactone (1 mg/Kg/d), catopril (1 mg/Kg/d), ibuprofen (30 mg/Kg/d) for oral and intravenous fluid therapy were given;<br>DOL 3: Intravenous therapy was replaced by oral KCl solution (10 mmol/Kg/d);<br>DOL 7: The boy left the hospital with the therapy of KCl and increased fluid intake with age	Growth retardation and dental enamel dysplasia
57	Zuo et al. [66]	Case series	Case 1 of 5	Combination therapy of indomethacin and potassium chloride supplementation, with daily dosage of 25 mg and 1.5 g, respectively	Diabetes insipidus, polyuria, and nocturnal enuresis
			Case 2 of 5	Vitamin D3	Nephrocalcinosis
			Case 3 of 5	Indomethacin	Growth retardation, polydipsia, and polyuria
			Case 4 of 5	Indomethacin	Gross hematuria
			Case 5 of 5	Indomethacin	Premature birth and severe electrolyte disturbance
